# Career Planning in Elite Soccer: The Mediating Role of Self-Efficacy, Career Goals, and Athletic Identity

**DOI:** 10.3389/fpsyg.2021.694868

**Published:** 2021-07-20

**Authors:** Ricardo Monteiro, Diogo Monteiro, Miquel Torregrossa, Bruno Travassos

**Affiliations:** ^1^Department of Sport Sciences, Universidade da Beira Interior, Covilhã, Portugal; ^2^ESECS, Polytechnic of Leiria, Leiria, Portugal; ^3^CIDESD, Research Center in Sports Sciences, Health Sciences and Human Development, Covilhã, Portugal; ^4^Departament de Psicologia Clínica i de la Salut, Universitat Autònoma de Barcelona, Barcelona, Spain; ^5^Portugal Football School, Portuguese Football Federation, Oeiras, Portugal

**Keywords:** career planning for life after sport, social cognitive factors, soccer players, career management, athletic identity, self-efficacy

## Abstract

This study proposed a model to examine the role of self-efficacy, career goals, and athletic identity (AI) on the career planning of elite soccer players. Two hundred and eighty-one elite soccer players (males) participated in this study. Means, SD, and bivariate correlations were calculated for the variables under analysis. The hypothesized effect of self-efficacy, career goals, and AI on career planning was tested using structural equation modeling (SEM). Results supported the adequacy of the proposed model in explaining the career planning. Career planning is positively influenced by the level of self-efficacy of players through the definition of career goals and is negatively influenced by the level of AI. At the same time, the self-efficacy of players through the definition of career goals positively influenced AI. These findings reinforce the important role of self-efficacy and career goals for the development of AI and career planning and at the same time the opposite relationship between AI and career planning. Thus, it is suggested that a balance on AI, maintaining high levels of self-efficacy and career goals, is required to improve the process of career planning and retirement.

## Introduction

Understanding the career of players through the umbrella of the Holistic Athletic Career Model (Wylleman, 2019) underlines a holistic life span and “beginning-to-end” approach of the career development of athletes, describing career pathways and predicting normative transitions between adjacent stages in a multilayer process. The predictability of normative transitions, such as career retirement, creates an opportunity to prepare athletes to cope with them in advance (Stambulova et al., 2009). In fact, the career of players is a process that should be prospectively managed to balance individual resources and barriers to prepare a smooth retirement and the life after sport (Alfermann and Stambulova, 2007; Demulier et al., 2013). In line with the social cognitive career theory (Lent and Brown, 2013), a positive relationship among self-efficacy, career goals, and career planning seems to be key individual resources to such processes (Demulier et al., 2013).

For instance, the self-efficacy of athletes has been one of the most important internal factors reported to explain successful career transitions (Wendling and Sagas, 2020). Self-efficacy could be defined as the belief or ability of an individual to perform a specific task or behavior to bring forth the desired outcome (Bandura, 2004). According to Lent and Brown (2013), self-efficacy in career transitions refers to the ability to manage specific tasks necessary with the preparation of such transitions, adjustments, or changes, i.e., the tasks related to career planning and management. In its turn, influenced by self-efficacy levels, career goals are defined as the intentions of athletes to be engaged with a certain task to achieve a specific outcome. Thus, career goals influenced by self-efficacy levels contribute to a better definition of career objectives and the consequent steps or planning to achieve them. More precisely, career goals refer to the definition of the intended set of professional, life, and after sport life achievements, the subsequent plans to achieve them, and to face the expected problems during the process (Creed et al., 2013; Demulier et al., 2013). When objectives are clearly identified, career plans are made to pursue those previously identified objectives, triggering career achievements and facilitating transitions. Career planning is defined as the definition of the actions or behaviors needed for career development. This kind of task, specific to career management, seems to be a crucial aspect in the context of sports career termination (Rogers and Creed, 2011). Individuals with high self-efficacy tend to better define career goals and plan their careers (Lent and Brown, 2013). In this regard, it is reasonable to assume that definitions of self-efficacy and goals are proximal antecedents of career planning (Demulier et al., 2013). Besides the theoretical point of view among the variables, several empirical studies have been shown the associations among self-efficacy, career goals, and career planning (e.g., Wendling and Sagas, 2020), which reinforces the importance of these variables in career transitions.

Previous study also pointed out that individual differences and the level of consciousness (Rogers et al., 2008) about career transitions and particularly the retirement have implications to the level of career planning and consequently to the retirement. In line with that, the level of athletic identity (AI) is another important internal factor that could contribute to explain career planning through variations in the consciousness about career development and retirement (Martin et al., 2014). High levels of AI may lead to an over-involvement and commitment in sports practice, with a reduction of attention to other social and career aspects, consequently decreasing the consciousness about career management and development (Cecić-Erpič et al., 2004). Variations in emotional stability associated with career choice, humor disorders, substance abuse, unrealistic expectations regarding a sports-related career, and a lack of preparation for life and work outside of the sports world were also the consequences related to the high levels of AI (Brown et al., 2000; Lally and Kerr, 2005; Stambulova and Ryba, 2014). In fact, a previous study with Olympic athletes revealed that athletes with strong AI tend to not plan career retirement with consequences for life after sports (Torregrosa et al., 2015). The intensity of AI varies in association with the past relationship to sports and current athletic experiences, as well as the personal experiences of athletes with athletic failures and successes (Horton and Mack, 2000). However, this study also revealed that athletes with higher AI tend to reveal higher levels of self-efficacy, particularly in relation to future career development related to the sport (Cabrita et al., 2014).

**FIGURE 1:**
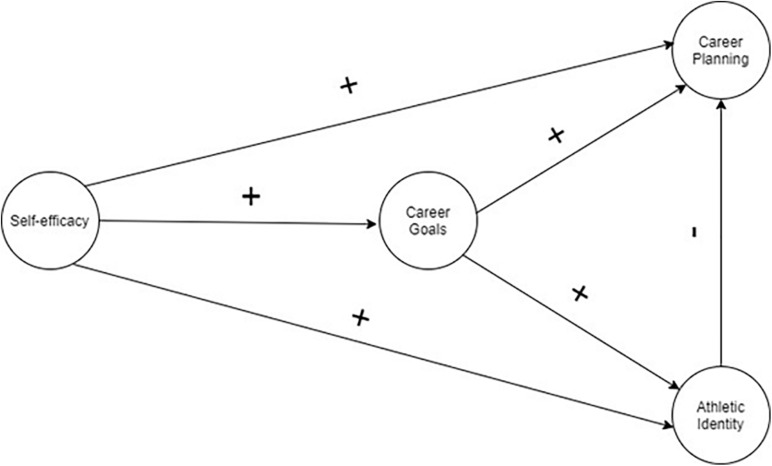
Hypothesized model to examine the role of self-efficacy, career goals, and athletic identity on career planning of elite soccer players.

Despite the previous study in the context of sport revealed that career planning is influenced by the levels of self-efficacy, AI, career goals, consciousness, competence, or even some personality traits (Demulier et al., 2013; Cabrita et al., 2014; Wendling and Sagas, 2020), to the best of our knowledge, any study analyzed the role of self-efficacy, career goals, and AI on career planning. This study proposed a model to examine the role of self-efficacy, career goals, and AI on the career planning of professional soccer players (see Figure 1). It is hypothesized that (a) self-efficacy positively predicted career goals, career planning, and AI, (b) career goals positively predicted career planning and AI, and (c) due to narrow focus in sports practice and despite the positive relationship with self-efficacy and career goals, it was expected that AI negatively predicted career planning.

## Materials and Methods

### Participants

A total of 281 active professional Portuguese soccer players (all males) aged between 18 and 39 years (*M* = 26.23; SD = 4.96), with a mean of 18.85 ± 2.32 years of practice, from several first and second professional league clubs in Portugal were recruited to participate in this study.

### Instruments

The instruments (i.e., Self-Efficacy Scale-Short Form, Career Goal Setting Scale, and Career Planning Scale) previously not validated in Portuguese were translated to Portuguese and back-translated to the original scale (Beaton et al., 2000). In addition, the factor structure of each scale was examined before testing the hypothesized model.

#### Self-Efficacy

The Self-Efficacy Scale-Short Form (Betz et al., 1996) was used to measure the self-efficacy of athletes. This scale comprises 12 items grouped into 1 factor (e.g., “select career options that best match your interests, values, skills, personality, and lifestyle”), which are preceded by the following sentence: “How confident are you about.” Athletes responded to each item using a 5-point Likert scale varying from 1 (no confidence) to 5 (total confidence). A confirmatory factor analysis (CFA) of this scale provided an acceptable fit to the data as follows: [χ^2^ = 354.92, DF = 54, SRMR = 0.021, Bollen–Stine (B–S) *p* = 0.032, Standardized Root Mean Square Residual and Root Mean Square Error of Approximation (RMSEA) = 0.076 (90% CI = 0.019, 0.143), Tucker–Lewis Index (TLI) = 0.969, and Comparative Fit Index (CFI) = 0.990].

#### Career Goals

Career Goal Setting scale (Wendling and Sagas, 2020) was used to assess the career goals of athletes. This scale is composed of five items grouped into one factor (e.g., “I have a clear set of goals for my career after sport”), preceded by the stem: “About my career goals …” Athletes responded to each item using a 7-point Likert scale, varied from 1 (totally disagree) to 7 (totally agree). The CFA exhibited the following fit: [χ^2^ = 91.30, DF = 5, SRMR = 0.025, B–S *p* = 0.019, RMSEA = 0.070 (90% CI = 0.052, 0.088), TLI = 0.958, and CFI = 0.986].

#### Career Planning

The Career Planning Scale (Wendling and Sagas, 2020) was used to measure the career planning of athletes after sport. This scale is composed of eight items, grouped into one factor (e.g., “I have a good understanding of the steps I need to take to reach my career goals”), preceded the stem: “About my career planning …” Athletes responded to each item using a 7-point Likert scale, which fluctuates from 1 (totally disagree) to 7 (totally agree). The items 1, 6, and 7 are reverse-coded. The CFA revealed an adequate fit to the data as follows: [χ^2^ = 72.46, DF = 20, SRMR = 0.018, B–S *p* = 0.218, RMSEA = 0.054 (90% CI = 0.001, 0.126), TLI = 0.987, CFI = 0.996].

#### Athletic Identity

The Portuguese version of the Athletic Identity Measurement Scale (AIMS-plus Portuguese version) (Cabrita et al., 2014) was used to assess the AI in line with the original AIMS (Brewer and Cornelius, 2001) and AIMS-plus (Cieslak et al., 2005). This questionnaire is comprised of 15 items distributed throughout 5 subscales, namely, social identity, exclusivity, self-identity, positive affectivity, and negative affectivity. The questionnaire contains a sliding-scale answer format, oscillating from 0 (totally disagree) to 10 (totally agree), thus, partial scores may be calculated for each dimension along with a total AI score. Higher scores indicate a greater level of identification with the role of athletes. The CFA revealed an adequate fit to the data as follows: [χ^2^ = 704.99, DF = 90, SRMR = 0.033, B–S *p* = 0.118, RMSEA = 0.046 (90% CI = 0.001, 0.092), TLI = 0.931, and CFI = 0.943].

### Data Collection

Written informed consent was obtained from soccer players. Approval for this study was granted by the Research Center in Sports Sciences, Health Sciences and Human Development (CIDESD), the institution that is registered in the Portuguese National Science Foundation (FCT) under the reference UID04045/2020. All procedures were under the 2013 Helsinki declaration and its later amendments.

The data were collected during the period from March to September 2020. Each athlete received an e-mail with a link to access the questionnaires using the Survey Monkey platform. Participants filled the multi-section survey in approximately 15 min.

### Data Analysis

Descriptive statistics were calculated for all variables under analysis. A two-step approach via the maximum likelihood estimator method (ml) was employed in Amos 23.0 IBM SPSS. (a) CFA was conducted to test the psychometric properties of the measurement model. Convergent validity, through average variance extracted (AVE), was calculated, and scores ≥0.50 were considered adequate (Hair et al., 2019). Discriminant validity was verified when the average variance factor of each factor was less than or equal to the square correlations across each construct underlying the measurement model (Hair et al., 2019). Finally, the composite reliability of each construct was calculated via the formula suggested by Raykov et al. (2016), and scores ≥0.70 were considered acceptable. (b) Structural equation modeling (SEM) to test the model fit was performed. In addition, standardized direct and indirect effects on the outcome variables were analyzed. Therefore, bootstrap resampling (1,000 samples) via bias-corrected 95% CI was used to assess the significance of the direct and indirect effects. The magnitude of effects was evaluated through the following suggestions by Cohen (2013): trivial (0–0.19), small (0.20–0.49), medium (0.50–0.79), and large (0.80 and greater). The traditional incremental and absolute goodness-of-fit indexes are as follows: CFI, TLI, RMSEA, and its respective CI (90%) were employed to test the model fit for both CFA and SEM, and the cut-off values suggested by Hair et al. (2019) were assumed as follows: CFI and TLI ≥ 0.90 and SRMR and RMSEA ≤ 0.08.

## Results

A prior analysis showed no missing values, neither univariate of outliers nor multivariate emerged. The values of skewness and kurtosis (i.e., between −2 and +2 and −7 and +7, respectively) revealed no deviations from univariate normality. However, the Mardia coefficient for multivariate kurtosis exceeded the expected value (>0.5). Thus, Bollen–Stine bootstrapping (2,000 samples) was employed for subsequent analysis. Possible collinearity diagnosis was tested by the variance inflation factor (VIF) and the tolerance test assuming values <10 for VIF and <0.01 for tolerance test. Consequently, the results displayed that both in VIF and tolerance tests scores were below 10 and above 0.1, respectively, guaranteeing the proper conditions to test the regression model.

### Descriptive Statistics

Descriptive results revealed high values (above midpoint) for all studied variables, and bivariate correlations exhibited a significant pattern across all variables. The highest correlation value was observed between career goals and career planning (*r* = 0.87), while the lowest values were observed between self-efficacy and AI (*r* = −0.29) (Table 1).

**TABLE 1:**
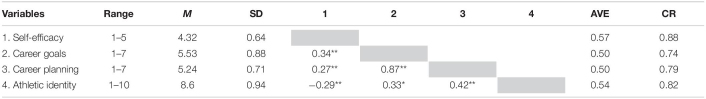
Descriptive statistics, bivariate correlations, average variance extracted, and composite reliability coefficients.

*M*, mean; SD, standard deviation; AVE, average variance extracted; CR, composite reliability; *p < 0.05; **p < 0.01.

### Measurement Model

The analysis of the measurement model included self-efficacy, career goals, career planning, and AI. Results showed the measurement model fit to the data as follows: χ^2^ = 1,927.79 (734), SRMR = 0.069, B–S *p* ≤ 0.001, RMSEA = 0.059 (90% CI = 0.047, 0.070), TLI = 0.922, and CFI = 0.935). Additionally, the measurement model revealed no problems of convergent (all factors presented scores above 0.50 in AVE). In terms of discriminant validity, a slight problem was identified involving the career goals and career planning factors, due to the square correlation between them are above the AVE of each factor. The remaining factors did not present discriminant validity problems. In addition, all of the constructs present an adjusted value of composite reliability since all of them are ≥0.70. Therefore, the psychometric properties of the measurement model were guaranteed (Table 1).

### Structural Model

The results from structural model displayed a good fit to the data as follows: [χ^2^ = 1,927.79 (734), SRMR = 0.049; B–S *p* ≤ 0.001, RMSEA = 0.042 (90% CI = 0.032, 0.052), TLI = 0.959, and CFI = 0.965].

Overall significant associations were observed among variables under analysis, specifically (a) self-efficacy displayed a positive and significant effect on career goals, AI, and career planning, (b) career goals displayed a positive and significant effect with career planning and AI, and (c) AI displayed a negative and significant effect with career planning.

Self-efficacy displayed an indirect positive and significant effect with career planning and AI via career goals. Career goals also displayed a positive and significant effect with career planning via AI. The observed effects varied from trivial to large (Table 2).

**TABLE 2:** Direct and indirect regression paths.

Regression path	Direct			Regression path	Indirect		
	β	95% CI	*p*		β	95% CI	*p*
SE → CG	0.24	[0.132; 0.388]	0.001	SE → CP	0.16	[0.091; 0.188]	0.002
SE → AI	0.22	[0.127; 0.334]	0.001	SE → AI	0.35	[0.242; 0.440]	0.001
SE → CP	0.41	[0.263; 0.563]	0.001	CG → CP	0.18	[0.063; 0.347]	0.001
CG → CP	0.83	[0.626; 0.881]	<0.001	–	–	–	–
CG → AI	0.28	[0.162; 0.425]	0.001	–	–	–	–
AI → CP	–0.32	[−0.468; −0.203]	0.002	–	–	–	–

*SE, self-efficacy; CG, career-goals; CP, career planning; AI, athletic identity; β, standardized coefficient; 95% CI, confidence interval at 95%; *p*, level of significance.*

## Discussion

The aim of this study was to test a model examining the role of self-efficacy on career goals and AI on career planning of elite soccer players. It was a first attempt to integrate AI with self-efficacy and career goals to further explain the process of career planning. In general, the model revealed a good fit to data, helping to reinforce the positive contribution of self-efficacy and career goals and the negative contribution of AI to the career planning of professional soccer players.

More specifically, the results confirmed all the hypotheses, revealing that (a) self-efficacy positively predicted career goals, career planning, and AI, (b) career goals positively predicted career planning and AI, and (c) AI negatively predicted career planning. Thus, while self-efficacy and career goals acted as mediating facilitators (Creed et al., 2013; Demulier et al., 2013; Wendling and Sagas, 2020), the AI played as a mediating barrier for career planning.

In this study, we aimed to look into the individual resources that positively or negatively constraint the process of career planning (Lent and Brown, 2013; Wendling and Sagas, 2020). Looking into each variable in the analysis, the results revealed that soccer players with higher self-efficacy seem to better define career goals and directly and indirectly career planning. The self-efficacy of players seems to contribute to a better definition of objectives of career and consequently to clarify their career plans for the future (Creed et al., 2013; Lent and Brown, 2013). Such results are in line with a previous study that showed that the definition of career goals of players was positively related to the quality of career retirement (Park et al., 2013; Debois et al., 2015). Furthermore, career planning seems to be influenced by the level of self-efficacy of players through the definition of career goals and building plans, helping to trigger the confidence of players in career self-management and decision-making. In fact, as previously pointed out by Monteiro et al. (2020), more than only the definition of goals, the achievement of goals demonstrated strong relation with the age of career retirement and also the level of retirement. However, the most important issue is not to achieve a high level of performance but to maintain such level over the career. The feeling of self-achievement in these cases seems to be crucial to maintain high levels of self-efficacy and a better definition of career planning (Demulier et al., 2013; Torregrosa et al., 2015).

Looking into the relationship among self-efficacy, career goals, and AI, according to our model, the results revealed that players with high self-efficacy revealed a better definition of career goals and directly and indirectly increased the level of AI. The results were in line with a previous study that revealed that players with a strong self-efficacy tend to reveal a strong sense of AI (Lamont-Mills and Christensen, 2006). During athletic career, high levels of AI contributed to soccer players exclusively maintain the focus on sports performance and on the management of professional activities (e.g., training seasons, game schedule, request rest, and pregame stages) (Torregrosa et al., 2015). That is, with the increase in self-efficacy and achievement of goals, soccer players become more confident and tend to develop their thinks and feels around the sports world (Creed et al., 2013; Demulier et al., 2013; Wendling and Sagas, 2020). Also, the level of AI seems to be positively associated with athletic performance, a desirable quality encouraged by coaches and structure of clubs (Martin et al., 2014). However, the major issue is that, as previously demonstrated, at the moment of career retirement, the high levels of AI tend to negatively impact the process and quality of retirement (Park et al., 2013).

In line with that, and according to our expectations, the results highlighted a negative relationship between AI and career planning. As observed with Olympic athletes (Torregrosa et al., 2015), soccer players (Carapinheira et al., 2019), handball players (Ekengren et al., 2018), and student athletes (Lally and Kerr, 2005; Wendling and Sagas, 2020), a strong AI is related to a lack of career planning. The behavior of elite athletes, and particularly the elite soccer players, to delay “undressing the sports sweater” could be related to the need to maintain the financial support and social status (Demulier et al., 2013; Martin et al., 2014) and to the lack of self-awareness and planning about the advantage to prepare the other career when the sports ends. Moreover, Cabrita et al. (2014) showed that AI could constraint the choices or opportunities for post sports career, being this a primary determining factor in whether an athlete chooses a sport-related career.

According to our model, the career goals could act as a mediator variable among sell efficacy, AI, and career planning. That is, the definition and management of objective and achievable career goals for the development and also to the end of career could ensure a more balanced and planned career development and retirement. Particularly, the identification and link between career goals and life goals could be assumed of particular interest to decrease the level of AI, promoting the process of career planning and career retirement (Torregrosa et al., 2015; Stambulova et al., 2020). Despite the lack of time and energy that athletes need to engage in non-sports-related activities (López de Subijana et al., 2015; Ekengren et al., 2018; Stambulova and Wylleman, 2019), it seems a key issue to decrease the level of AI and consequently allows a better career planning process. Recently, the International Society of Sport Psychology assumed that to improve the AI foreclosure in athletes, there is a need to create new possibilities for the self-exploration of multidimensional personal identities and the development of a dual career of athletes (Stambulova et al., 2020). Thus, based on our results and in line with the holistic athletic career model (Wylleman et al., 2004), it is suggested that the career management of elite athletes should start with the definition of career goals through the definition of some key performance indicators that help soccer players and managers to identify the achievement of goals (Monteiro et al., 2020) followed by the definition of parallel life goals and exploration of a dual career perspective in order to decrease AI and improve the career planning (Torregrosa et al., 2015).

In summary, the proposed model demonstrated the mediating positive influence that career goals have on the relationship among self-efficacy, career planning, and AI. These findings reinforce the important role of self-efficacy in this process and reinforce findings about the decision-making process on sports career retirement (Park et al., 2013). Although this study contributes to the understanding of the role of self-efficacy, career goals, and career planning as well as AI in active soccer players, it has some limitations. All the variables were assessed at the same time, so we can only address the associations among the variables without determining causality. In this sense, longitudinal or experimental studies are necessary to further examine the effects of the studied variables. In addition, future studies should try to examine the respective effects of each studied variable and possibly to examine whether these effects are invariant for soccer players of different genders and different ages. It is possible that studied variables may play different roles for male or female players or that their effects may fluctuate according to the variations in their educational, social, or financial status. Finally, in this study, we included active soccer players, and future studies should also explore other sports (e.g., futsal) and assess the perspective of soccer players after their career retirement.

Despite the positive interactions with self-efficacy and career goals, AI revealed a negative relationship with career planning. Thus, managers, coaches, and sports psychologists should start since the beginning of careers of soccer players to encourage the definition of realistic career and life goals as well as the promotion of a dual career project that allows decreasing the level of AI of players. The definition of career goals and the integration of life goals in the process of career management seems to be crucial for career planning and consequently for the process of retirement.

## Data Availability Statement

The raw data supporting the conclusions of this article will be made available by the authors, if required and with some restrictions considering its individual nature.

## Ethics Statement

The studies involving human participants were reviewed and approved by Ethics Committee by the Research Center in Sports Sciences, Health Sciences and Human Development (CIDESD). The patients/participants provided their written informed consent to participate in this study.

## Author Contributions

RM, DM, MT, and BT contributed to the conception and design of the study. RM and DM conducted the statistical analyses and wrote the first draft of the manuscript. MT and BT wrote several sections of the manuscript. All the authors contributed to the manuscript revision, and read and approved the submitted version of the manuscript.

## Conflict of Interest

The authors declare that the research was conducted in the absence of any commercial or financial relationships that could be construed as a potential conflict of interest.
